# Toward a Phage Cocktail for Tuberculosis: Susceptibility and Tuberculocidal Action of Mycobacteriophages against Diverse Mycobacterium tuberculosis Strains

**DOI:** 10.1128/mBio.00973-21

**Published:** 2021-05-20

**Authors:** Carlos A. Guerrero-Bustamante, Rebekah M. Dedrick, Rebecca A. Garlena, Daniel A. Russell, Graham F. Hatfull

**Affiliations:** a Department of Biological Sciences, University of Pittsburgh, Pittsburgh, Pennsylvania, USA; Weill Cornell Medical College

**Keywords:** *Mycobacterium tuberculosis*, bacteriophage therapy, bacteriophages, tuberculosis

## Abstract

The global health burden of human tuberculosis (TB) and the widespread antibiotic resistance of its causative agent Mycobacterium tuberculosis warrant new strategies for TB control. The successful use of a bacteriophage cocktail to treat a Mycobacterium abscessus infection suggests that phages could play a role in tuberculosis therapy. To assemble a phage cocktail with optimal therapeutic potential for tuberculosis, we have explored mycobacteriophage diversity to identify phages that demonstrate tuberculocidal activity and determined the phage infection profiles for a diverse set of strains spanning the major lineages of human-adapted strains of the Mycobacterium tuberculosis complex. Using a combination of genome engineering and bacteriophage genetics, we have assembled a five-phage cocktail that minimizes the emergence of phage resistance and cross-resistance to multiple phages, and which efficiently kills the M. tuberculosis strains tested. Furthermore, these phages function without antagonizing antibiotic effectiveness, and infect both isoniazid-resistant and -sensitive strains.

## INTRODUCTION

Mycobacterium tuberculosis, the causative agent of human tuberculosis, has plagued humanity for nearly 9,000 years, with the earliest written records of the disease going back more than 3,000 years in India and China (1). With the advent of antibiotics such as streptomycin and isoniazid, the end of tuberculosis has been heralded since the late 1950s and early 1960s (2, 3). Unfortunately, since the 1990s there has been a resurgence of tuberculosis worldwide and the emergence of multiple drug-resistant (MDR), extensively drug-resistant (XDR), and totally drug-resistant (TDR) strains of M. tuberculosis (4, 5). The lengthy treatment duration, combined with adverse side effects and the relative high cost in developing countries, has resulted in poor compliance with treatment regimens, further fueling the emergence of drug-resistant strains (6). New antibiotics, including bedaquiline (7), have been developed, but the need for new therapeutic strategies is clear (8).

Bacteriophages are viruses that infect bacterial hosts and are the most abundant organisms on the planet (9, 10). They are genetically diverse with large proportions of genes having no close relatives in extant GenBank entries (11). More than 2,000 individual mycobacteriophages, viruses that infect Mycobacterium spp. have been isolated and sequenced (https://phagesdb.org), mostly within the Science Education Alliance Phage Hunters Advancing Genomics and Evolutionary Science (SEA-PHAGES) program (12). These phages have been organized according to their overall sequence relationships into 29 genomic groups designated clusters A through Z and AA to AC. Some clusters are sufficiently diverse to warrant division into subclusters; for example, cluster A contains 20 subclusters (A1 to A20). In addition, there are currently nine “singleton” (sin) mycobacteriophages, each with no close relative (13).

Bacteriophages infecting M. tuberculosis were first isolated in the 1950s (14) and have been used to type clinical isolates (15). Four major subtypes of M. tuberculosis (A, B, C, and I) have been described (16, 17), each of which differs in their phage susceptibility profiles (18). These early typing studies noted the association of M. tuberculosis phage types with particular human populations and geographical origins (16, 19), and reported that different phage types exhibit various levels of virulence (20). However, little was known about the genetic relationships of these typing phages and many are now lost or unavailable. Taking advantage of a larger mycobacteriophage collection, genomic information, and host range analyses of 220 mycobacteriophages showed a close relationship between cluster designation and host range. Specifically, subcluster A2, A3, K1, K2, K3, K4, and G1 phages are able to infect M. tuberculosis mc^2^7000, an avirulent derivative of M. tuberculosis H37Rv (21, 22). However, this phage collection has expanded considerably since these analyses were reported in 2012 (12).

The M. tuberculosis complex (MTBC) includes Mycobacterium africanum, Mycobacterium canettii, Mycobacterium bovis, Mycobacterium microti, Mycobacterium orygis, Mycobacterium caprae, Mycobacterium pinnipedii, Mycobacterium suricattae, and Mycobacterium mungi, in addition to M. tuberculosis (23). These are obligate pathogens that cause tuberculosis and tuberculosis-like infections in humans and animals and likely diverged from a common ancestor in Africa during the Neolithic age (24). The human-adapted strains can be grouped into nine distinct lineages found in different parts of the world (25). Lineages L1, L2, L3, L4, and L7 are M. tuberculosis *sensu stricto*, and L5, L6, and L9 are M. africanum (23, 25). Lineages L2 and L4 are widespread (26), with L2 predominating in Asia and L4 being the most common lineage found in Africa, Europe, and the Americas (27, 28). Lineages L1 and L3 are found in South Asia and Africa near the Indian Ocean and L7 is restricted to Ethiopia (23). The M. africanum lineages L5 and L6 are only found in western Africa, and account for as many as 50% of the cases of tuberculosis in that region (23). Lineages L8 and L9 have been recently described and are very rare. L8 is thought to have diverged early from the common ancestor of the human-adapted M. tuberculosis complex (29); L9 (also M. africanum) is closely related to L6, but is only found in eastern Africa. Epidemiological studies suggest that lineages 2 and 4 may be more virulent than lineages 1 and 3 (30), and lineage 2 strains are commonly drug resistant (28). Additionally, lineages 2 and 4 may be readily transmissible, although the molecular bases are unclear (19, 27, 30).

Bacteriophages have been used to treat a variety of bacterial infections, notably in the former Soviet Union and its successor states (31, 32). The first successful use of phages to treat a mycobacterial infection was in a 15-year-old with cystic fibrosis with a disseminated Mycobacterium abscessus infection after a bilateral lung transplant (33); a three-phage cocktail was administered intravenously without the emergence of phage resistance. The phages were identified by screening M. smegmatis phages for the small subset with host ranges that include M. abscessus, as few phages have been isolated using M. abscessus directly. However, most mycobacteriophages are temperate and two of the phages needed to be engineered to ensure lytic growth and efficient antimicrobial activity (33–35). Interestingly, there is substantial variation in phage susceptibility among clinical isolates of M. abscessus, and the cocktail used successfully in the one patient is not suitable for other patients (36). The complex and highly variable plasmid and prophage content may influence the phage infection profiles by expressing viral defense systems (36, 37). Nonetheless, the success of this intervention lends weight to the concept that there may be a role for phages in tuberculosis control (38). Prophylactic prevention of M. tuberculosis growth following phage aerosolization in mice offers further support (39).

The therapeutic potential of phages for treating tuberculosis has not been thoroughly explored, in part because relatively few phages are available. Thus, little is known about variation in susceptibility and killing of M. tuberculosis clinical isolates in different lineages, mechanisms of phage resistance, or interactions between phages and antibiotics. Moreover, the virulence, slow growth (24 h doubling time), and propensity for cellular clumping present substantial challenges to detailed phage investigations using M. tuberculosis. Here, we screened an expanded panel for new phages that infect M. tuberculosis, enhanced potentially useful phages by genome engineering and host range manipulation, and defined variations in phage infection in a suite of M. tuberculosis clinical isolates. By defining patterns and mechanisms of phage resistance and interactions with antibiotics, we have assembled a five-phage cocktail that efficiently kills all of the tested M. tuberculosis strains and which can be used to evaluate phage therapy for human tuberculosis.

## RESULTS

### Identification of phages infecting M. tuberculosis H37Rv.

Many sequenced mycobacteriophage isolates were shown previously to efficiently infect M. tuberculosis mc^2^7000 (an avirulent derivative of H37Rv), but they belong to a few clusters/subclusters (specifically A2, A3, G, K1, K2, and K3). Although phage BPs (cluster G1) does not efficiently infect mc^2^7000, host range mutants containing single amino acid substitutions in the tail gene (gene *22*) can be readily isolated (21, 35). Seven of twelve phages used previously in M. tuberculosis typing studies have recently been sequenced (40), four (DNA III, Clark, Sedge, and Legendre) are cluster G phages based on BLAST analysis of the published genomes; two (BK1 and GS4E) are in subclusters A1 and A2, respectively (40), and the seventh is the singleton M. tuberculosis-specific phage DS6A (41) (Table S1 in the supplemental material). The report that phage BK1 (subcluster A1) infects M. tuberculosis H37Rv (15, 42) is in sharp contrast to the finding that 24 subcluster A1 phages tested previously do not (21).

**FIG 1:**
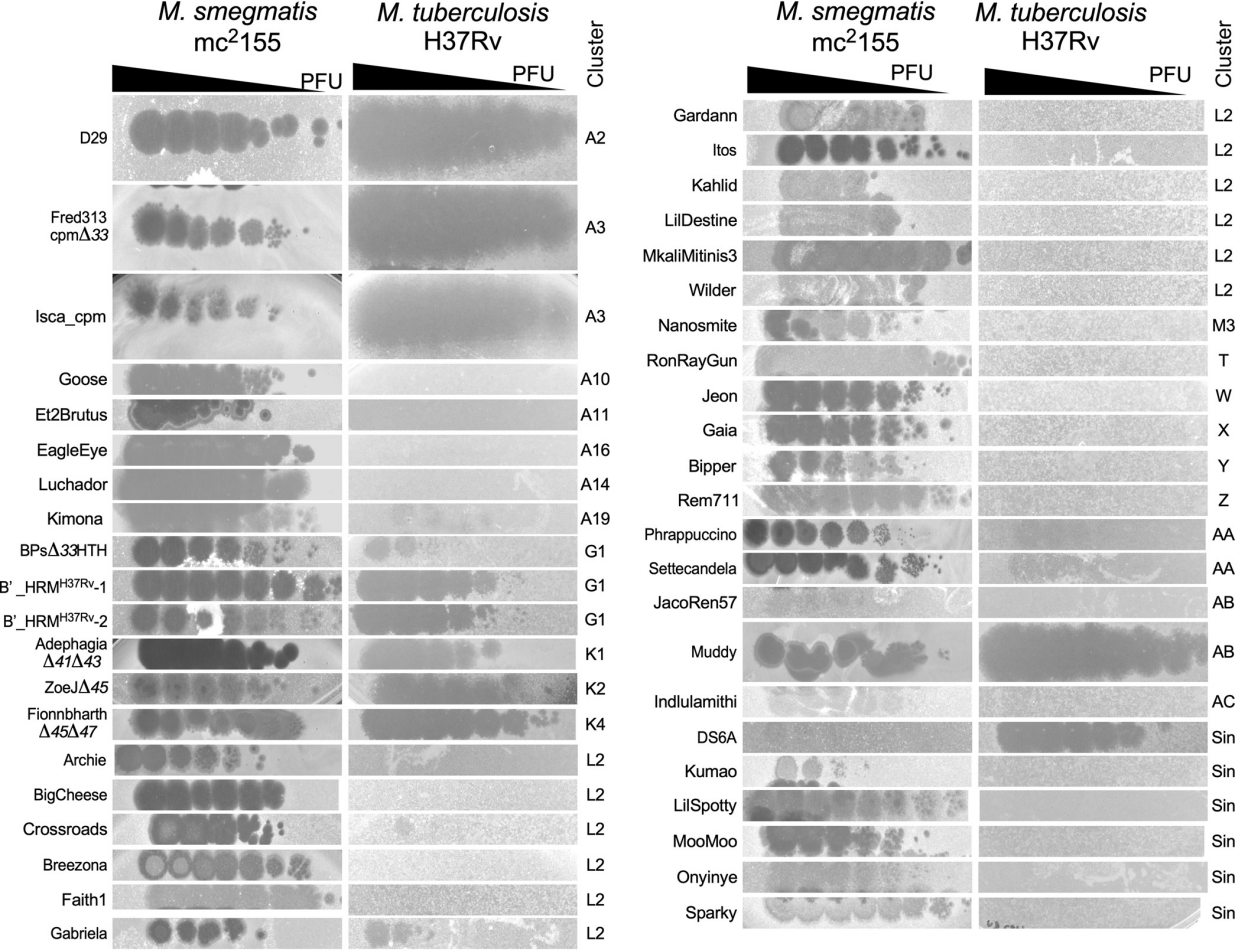
Phage susceptibility of M. tuberculosis H37Rv. Phage lysates, shown on the left, were 10-fold serially diluted and 3 μl of the 10^−1^ to 10^−8^ dilutions were spotted onto top agar overlays containing M. smegmatis mc^2^155 or M. tuberculosis H37Rv. Phage cluster/subcluster designation are shown on the right.

To further analyze the phage susceptibility of M. tuberculosis, we screened representatives of M. smegmatis clusters/subclusters identified since 2012 (Table 1) for their efficiency of plaquing (EOP) on virulent M. tuberculosis H37Rv relative to M. smegmatis mc^2^155, on which they were isolated (Fig. 1). We also included engineered lytic derivatives from temperate phages reported previously to infect M. tuberculosis (21), in which the repressor gene is removed (Fig. 1); for some of these (e.g., AdephagiaΔ*41*Δ*43* and FionnbharthΔ*45*Δ*47*) (Table 1) the integrase gene is also deleted. As reported previously (43), DS6A, a singleton, infects M. tuberculosis H37Rv but not M. smegmatis, but none of the other singleton M. smegmatis phages infect H37Rv (Fig. 1). However, phage Muddy (cluster AB) efficiently infects M. tuberculosis H37Rv and forms large clear plaques. Settecandela and Phrappuccino (both cluster AA) also infect H37Rv but are temperate and form extremely turbid plaques reflecting high lysogenization frequencies (Fig. 1); clear plaque variants of these phages have not yet been isolated. None of the other newly isolated phages (representatives of clusters T, M, W, X, Y, Z, and AC, subclusters A10, A11, A15, A16, and A19, and three singletons) efficiently infect M. tuberculosis H37Rv (Fig. 1). AdephagiaΔ*41*Δ*43*, ZoeJΔ*45*, and FionnbharthΔ*45*Δ*47* (subclusters K1, K2, and K4, respectively), D29 (A2), two host range mutants of BPs (BPsΔ*33*HTH_HRM^H37Rv^-1 and BPsΔ*33*HTH_HRM^H37Rv^-2, subcluster G1; Table 1) (21), and both subcluster A3 phages (Isca_cpm and Fred313_cpmΔ*33*, see below) also efficiently infect M. tuberculosis H37Rv (Fig. 1). The temperate phage Isca (A3) was originally isolated on M. abscessus strain GD01 (33), and here we use Isca_cpm, a naturally occurring lytic derivative in which the repressor gene is defective (36).

**TABLE 1:** Phages used in this study

Phage	Parent^*d*^	Cluster^*d*^	HRM^*a*^^,^^*d*^	Temperate^*b*^	Accession number^*c*^
Wild type					
D29	NA	A2	NA	No	AF022214
Phrappuccino	NA	AA	NA	Yes	MK937592
Settecandela	NA	AA	NA	Yes	MT114163
JacoRen57	NA	AB	NA	No	MK279840
Muddy	NA	AB	NA	No	KF024728
Indlulamithi	NA	AC	NA	No	MN585993
BigCheese	NA	L2	NA	Yes	MH834600
Itos	NA	L2	NA	Yes	MN703410
Archie	NA	L2	NA	Yes	KT591489
Breezona	NA	L2	NA	Yes	KC691254
Crossroads	NA	L2	NA	Yes	KF024731
Faith1	NA	L2	NA	Yes	JF744988
Gabriela	NA	L2	NA	Yes	MN703406
Gardann	NA	L2	NA	Yes	KX507361
Kahlid	NA	L2	NA	Yes	MN586052
LilDestine	NA	L2	NA	Yes	MH779511
MkaliMitinis3	NA	L2	NA	Yes	KU234099
Wilder	NA	L2	NA	Yes	KX580962
Nanosmite	NA	M3	NA	Yes	MW578836
RonRayGun	NA	T	NA	Yes	KM591905
Jeon	NA	W	NA	No	MH001450
Gaia	NA	X	NA	Yes	KJ567043
Bipper	NA	Y	NA	Yes	KU728633
Rem711	NA	Z	NA	yes	MG770216
DS6A	NA	Sin	NA	No	JN698994
Kumao	NA	Sin	NA	Yes	MG009575
LilSpotty	NA	Sin	NA	Yes	MK977707
MooMoo	NA	Sin	NA	Yes	MH001449
Onyinye	NA	Sin	NA	No	MN813687
Sparky	NA	Sin	NA	Yes	KM083128
Mutants					
Fred313_cpm	Fred	A3	NA	No	MF373840
Isca cpm	Isca	A3	NA	No	MN586063
Muddy_HRM^N0157^-1	Muddy	AB	gp24 G487W	No	KF024728
Muddy_HRM^N0157^-2	Muddy	AB	gp24 T608A	No	KF024728
Muddy_HRM^N0052^-1	Muddy	AB	gp24 E680K	No	KF024728
BPsΔHTH*33*	BPs	G1	NA	No	EU568876
BPsΔHTH*33_*HRM^H37Rv^-1	BPs ΔHTH*33*	G1	gp22 A599V	No	EU568876
BPsΔHTH*33_*HRM^H37Rv^-2	BPs ΔHTH*33*	G1	gp22 F280C	No	EU568876
AdephagiaΔ*41*Δ*43*	Adephagia	K1	NA	No	JF704105
FionnbharthΔ*45*Δ*47*	Fionnbharth	K4	NA	No	JN831653
CG-REM-1	Fionnbharth Δ*45*Δ*47*	K4	gp26 G93D	No	JN831653
CG-REM-2	Fionnbharth Δ*45*Δ*47*	K4	gp26 G93R	No	JN831653
ZoeJΔ*45*	ZoeJ	Κ2	NA	No	KJ510412

a HRM, host range mutant. Substitutions are shown for gene product (gp) with the specific amino acid changes.

b Temperate designation determined either experimentally or predicted by bioinformatics.

c GenBank accession numbers are shown. For mutants, the number of the parent phage is shown.

d NA, not available; sin, singleton (no cluster).

Screening of M. smegmatis phages for those that infect M. abscessus GD01 identified phages Itos and Gabriela (both in subcluster L2) as potentially having a broad host range (33). However, subcluster L2 phages also vary greatly in their response to prophage-mediated defense systems (44). We therefore selected a set of 12 different L2 phages to screen against M. tuberculosis H37Rv (Fig. 1). Most show no infection, although Gabriela infects at a reduced EOP (10^−3^). This is consistent with the report that the subcluster L2 phage Celfi infects M. tuberculosis mc^2^6230, a derivative of M. tuberculosis H37Rv (45). The genomic basis for these differences is unclear, as subcluster L2 genomes are very closely related to each other (44). Taken together, these data show that one or more phages within clusters/subclusters A2, A3, G1, K1, K2, K4, L2, AA, and AB and the singleton DS6A are able to infect M. tuberculosis H37Rv and are candidates for having therapeutic potential. It is striking that, with the exception of Muddy, all of these are temperate or lytic derivatives of temperate phages.

### Strain variation in phage susceptibilities.

**FIG 2:**
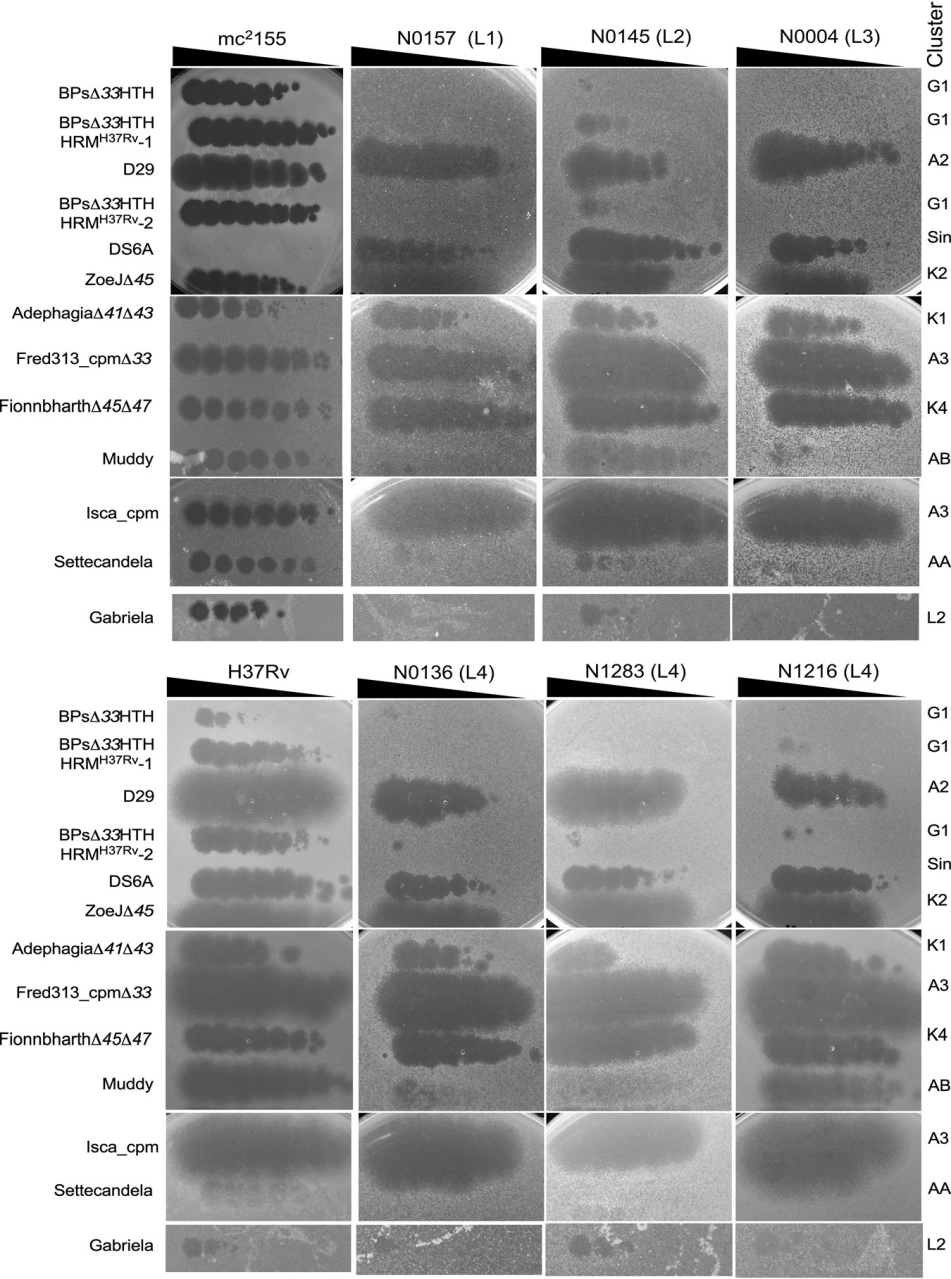
Phage infection of strains from different M. tuberculosis lineages. Phage lysates, as indicated on the left, were spotted onto lawns of M. smegmatis mc^2^155, M. tuberculosis H37Rv, and six M. tuberculosis clinical isolates. The lineage (i.e., L1, L2, L3, or L4) of each M. tuberculosis strain is shown in parentheses. A summary of phage infections of a larger panel of strains is shown in Table 3.

Unfortunately, the relationship between the historic phage types of M. tuberculosis and the contemporary genomic lineages is not known, although some assumptions could be made based on their geographical origin because MTBC members are highly sympatric (23). To explore phage susceptibility profiles of extant M. tuberculosis isolates, we obtained a set of reference strains with several representatives of lineages L1 to L6 (Table 2); all but one are part of the human MTBC reference set (46). Strain N0153 (L1), also known as T83, differs from its relative N0157 in its methylation pattern (47) and lacks the prophage-like element phiRv2 (9, 48). Sixteen strains were successfully propagated and together with M. tuberculosis H37Rv (L4) were tested for sensitivity to phages that infect M. tuberculosis H37Rv (Fig. 2, Table 3). These include at least three strains in lineages L1, L2, L3, and L4 belonging to M. tuberculosis *sensu stricto* and three members of M. africanum lineages L5/L6 (Table 2, Table 3), spanning the sublineage designations where known (Table 2).

**TABLE 2:** Mycobacterium tuberculosis strains used in this study

Strain	Parent	Lineage^*a*^	Sublineage^*a*^	Species	Mutations^*b*^	Comments^*c*^
H37Rv	NA	L4	4.10	M. tuberculosis	WT	
mc^2^4877	H37Rv	L4	NA	M. tuberculosis	*katG* del 371g	
N0157	NA	L1	L1.2.1	M. tuberculosis	WT	
N0072	NA	L1	L1.1.2	M. tuberculosis	WT	
N0153	NA	L1	NA	M. tuberculosis	WT	
N0145	NA	L2	L2.2.1.1	M. tuberculosis	WT	
N0052	NA	L2	L2.2.2	M. tuberculosis	WT	
N0031	NA	L2	L2.1	M. tuberculosis	WT	
N0155	NA	L2	L2.2.1	M. tuberculosis	WT	
N0004	NA	L3	NA	M. tuberculosis	WT	
N1274	NA	L3	NA	M. tuberculosis	WT	
N0054	NA	L3	NA	M. tuberculosis	WT	
N1216	NA	L4	L4.6.2.2	M. tuberculosis	WT	
N0136	NA	L4	L4.3.3	M. tuberculosis	WT	
N1283	NA	L4	L4.2.1	M. tuberculosis	WT	
N1063	NA	L5	NA	M. africanum	WT	
N0091	NA	L6	NA	M. africanum	WT	
N1202	NA	L6	NA	M. africanum	WT	
CG20	H37Rv	L4	NA	M. tuberculosis	C1939970Δ	Adephagia-R
CG21	H37Rv	L4	NA	M. tuberculosis	T1166874C	Fionnbharth-R
CG22	N1283	L4	NA	M. tuberculosis	ND	Adephagia-R
CG23	H37Rv	L4	NA	M. tuberculosis	Prophage frag	Fred313-R
CG24	H37Rv	L4	NA	M. tuberculosis	Prophage frag	Fred313-R
CG25	H37Rv	L4	NA	M. tuberculosis	Prophage frag	Fred313-R

a Strain lineages and sublineages are as reported in Borrell et al. (46). NA, not available.

b Mutations relative to the parent strain are shown. Prophage frag, integrated parts of phage; ND, not determined.

c Resistance to phages is denoted as Phage-R.

**TABLE 3:** Phage susceptibilities of M. tuberculosis strains^*a*^

Phage	Cluster	Lineage 1			Lineage 2				Lineage 3			Lineage 4				L5	Lineage 6	
		N0072	N0153	N0157	N0052	N0155	N0145	N0031	N0004	N1274	N0054	N1216	N0136	N1283	H37Rv	N1063	N0091	N1202
AdephagiaΔ*41*Δ*43*	K1	+++^1^	+++	+++	+++	+++	+++	+	+++	+++	+++	+++	+++	+++	+++	+++	+++	+
ZoeJΔ*45*	Κ2	ΝΤ	+++	+++	+++	+++	+++	ΝΤ	+++	ΝΤ	ΝΤ	+++	+++	+++	+++	+++	ΝΤ	ΝΤ
D29	A2	+++	+++	+++	+++	+++	+++	+	+++	+++	+++	+++	+++	+++	+++	+++	+++	NT
FionnbharthΔ*45*Δ*47*	K4	+++	+++	+++	+++	+++	+++	+++	+++	+++	+++	+++	+++	+++	+++	+	+++	+
Fred313_cpmΔ*33*	A3	+++	+++	+++	+++	+++	+++	−	+++	+++	+++	+++	+++	+++	+++	−	−	+
Muddy WT	AB	+	+	−	+++	+++	+++	−	+	+	+	+++	+	+	+++	+	−	+
Muddy_HRM^N0157^-1	AB	+++	+++	+++	+++	+++	+++	NT	+++	+++	+++	+++	+++	+++	+++	+	NT	+
Muddy_HRM^N0157^-2	AB	+++	+++	+++	+++	+++	+++	NT	+++	+++	+++	+++	+++	+++	+++	+	NT	+
Muddy_HRM^N0052^-1	AB	+++	+++	+++	+++	+++	+++	+++	+++	+++	+++	+++	+++	+++	+++	+	NT	+
DS6A	Sin	+++	+++	+++	+++	+++	+++	+	+++	+++	+++	+++	+++	+++	+++	+	+	NT
BPsΔHTH*33*	G1	NT	−	−	−	−	−	−	−	−	−	−	−	−	+	−	NT	NT
BPsΔHTH*33*_HRM^H37Rv^-1	G1	NT	−	−	−	−	+	NT	−	−	−	−	−	−	+++	−	NT	NT
BPsΔHTH*33*_HRM^H37Rv^-2	G1	NT	−	−	−	−	+	NT	−	−	−	−	−	−	+++	−	NT	NT
Isca_cpm	A3	NT	+++	+++	+++	+++	+++	NT	+++	ΝΤ	+++	+++	+++	+++	+++	+	NT	+
Settecandela	AA	NT	−	−	NT	NT	+++	NT	−	NT	NT	−	−	+	+++	−	NT	−
Gabriela	L2	NT	−	−	NT	NT	+	NT	−	NT	NT	+	−	+	+	−	NT	−

a The scoring system denotes efficiencies of plaquing relative to M. smegmatis as follows: +++, >0.1; +, infection seen at the highest titers plated, but EOP <10^4^; −, no infection. EOP for DS6A, which does not infect M. smegmatis, is relative to infection of M. tuberculosis H37Rv. NT, not tested.

The phage infection patterns of these strains have several notable features (Fig. 2, Table 3). First, most of the strains are infected by multiple phages, with the notable exception of N0031 (L2), which is only infected efficiently by FionnbharthΔ*45*Δ*47* (Fig. 2, Table 3). Second, some phages discriminate between strains, including Fred313_cpmΔ*33*, which does not efficiently infect N0031 (L2), N1063 (L5), or lineage 6 strains, and Muddy, which does not efficiently infect any L1, L3, L5, or L6 strains, N0031 (L2), or lineage 4 strains N0136 and N1283 (Fig. 2, Table 3); on some strains (e.g., N0145), Muddy plaques are noticeably more turbid than on H37Rv (Fig. 2), reflecting the phenotype observed on M. smegmatis (33). In addition, the BPs host range mutants (HRMs; BPsΔ*33*HTH_HRM^H37Rv^-1, and BPsΔ*33*HTH_HRM^H37Rv^-2) are strictly restricted to H37Rv infection, and do not efficiently infect any other strain (Fig. 2, Table 3).

### Host range mutants of phage Muddy.

**FIG 3:**
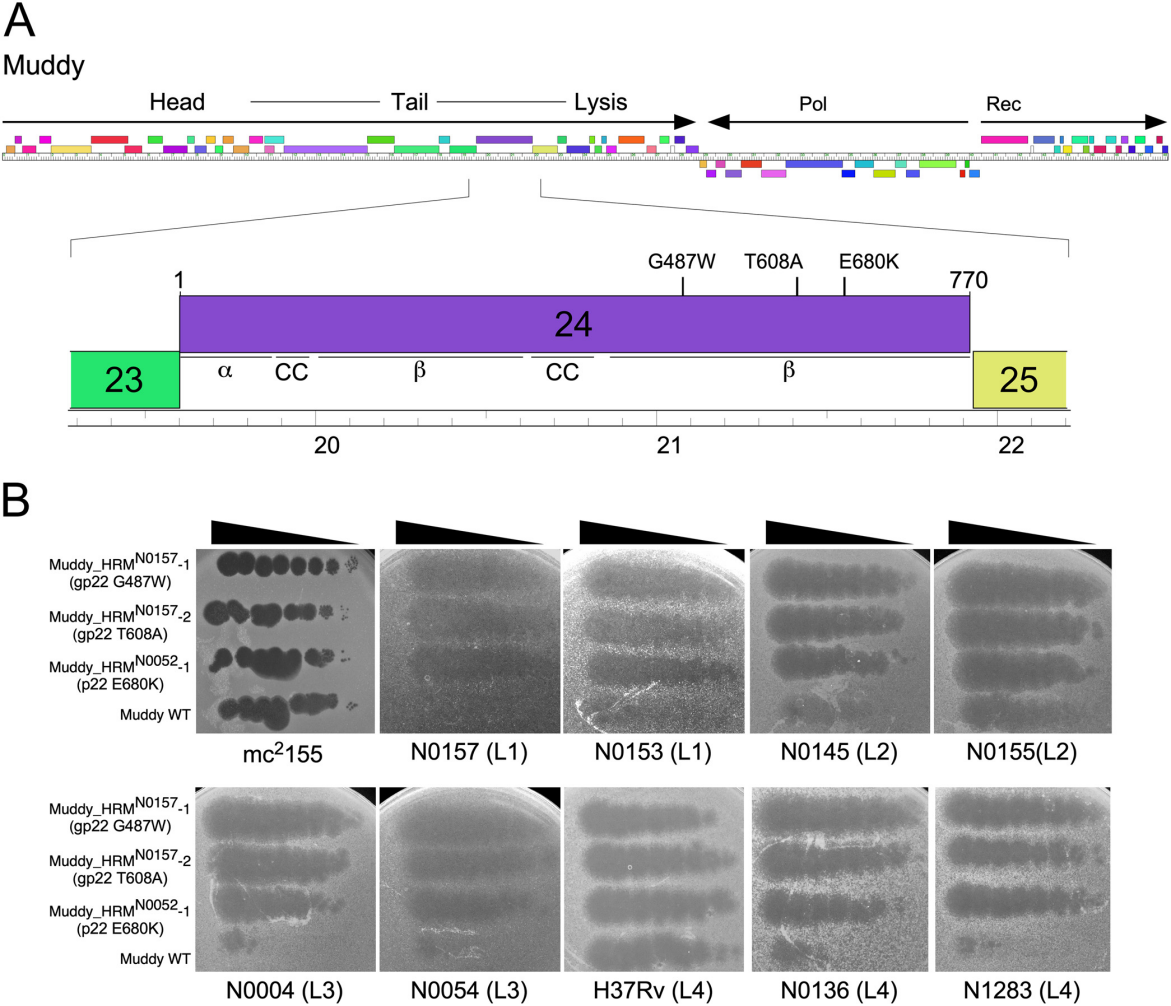
Expanded host range mutants of phage Muddy. (A) A map of the Muddy genome shows genes as colored boxes above a genome marker. The direction of transcription (horizontal arrows) and locations of head, tail, and lysis genes are indicated; DNA polymerase (Pol) and RecA (Rec) genes are also shown. Below is an expanded view of tail gene *24* showing predicted secondary structure motifs (α, alpha helix; β, beta sheets; CC, coiled coil). The positions of amino acid substitutions conferring an expanded host range phenotype are shown above gene *24*. (B) Lysates of WT Muddy and host range mutant derivatives (as shown) were serially diluted and spotted onto lawns of mycobacterial strains as indicated. The lineage of each M. tuberculosis strain is shown in parentheses.

Although Muddy poorly infects some M. tuberculosis strains, plaques were observed on several of these strains when high titers were plated. Plaques were picked from plating of Muddy on N0157 (L1) and N0052 (L2; from noticeably clear plaques at high titer), recovered on M. smegmatis, and further characterized. DNA sequence analysis (see below) showed the Muddy lysate derived from N0157 was a mixture of two phages carrying different mutations, which were separated and purified. Following purification, the three host range mutants (HRMs) were designated Muddy_HRM^N0157^-1, Muddy_HRM^N0157^-2, and Muddy_HRM^N0052^-1 (Table 1). All three mutants retain the ability to infect M. smegmatis, and lysates prepared on M. smegmatis efficiently infect the M. tuberculosis strain they were isolated on. Complete genome sequencing showed that all three derivatives have distinct single base changes in the putative tail gene *24* (G21064T, A21427G, and G21643A), conferring amino acid substitutions G487W, T608A, and E680K, respectively, all within a predicted extended β-sheet at the C terminus of the gp24 protein (Fig. 3A). All three HRMs infect all M. tuberculosis strains tested with an EOP of one relative to M. smegmatis, with the exception of Muddy_HRM^N0052^-1, which has a slight EOP reduction (∼10^−1^) on strains N0004 (L3), N0145 (L2), and N0136 (L4) (Fig. 3B, Table 3). The host range expansion conferred by these substitutions is impressive in broadening their infection to all of the other L1 to L4 strains tested (Fig. 3, Table 3), including infection of strain N0031 by Muddy_HRM^N0052^-1, which was otherwise only infected by FionnbharthΔ*45*Δ*47.*

Targeted PCR screening and sequencing of additional Muddy plaques picked from strains L0072 (L1), N0004 (L3), and N1283 (L4) showed that each had one of the same three substitutions in gp24. Plaques derived from strains N0072 and N0004 have the T608A and G487W substitutions, respectively, and plaques derived from N1283 had both the G487W and E680K mutations. Interestingly, although wild-type (WT) Muddy infects strain N1216 relatively well (Table 3), and without the turbidity observed for the L2 strains (e.g., N0145, Fig. 3), one out of eight plaques screened also had the E680K mutation. These three substitutions thus appear to be the primary changes capable of expanding the host range of Muddy to include all of the M. tuberculosis L1 to L4 strains tested here. For strain N1063 (L5), all three mutations confer some improvement in infection, but for strain N1202, WT Muddy and the mutants infect at similarly reduced efficiencies (Table 3).

### Phage resistance in M. tuberculosis.

**FIG 4:**
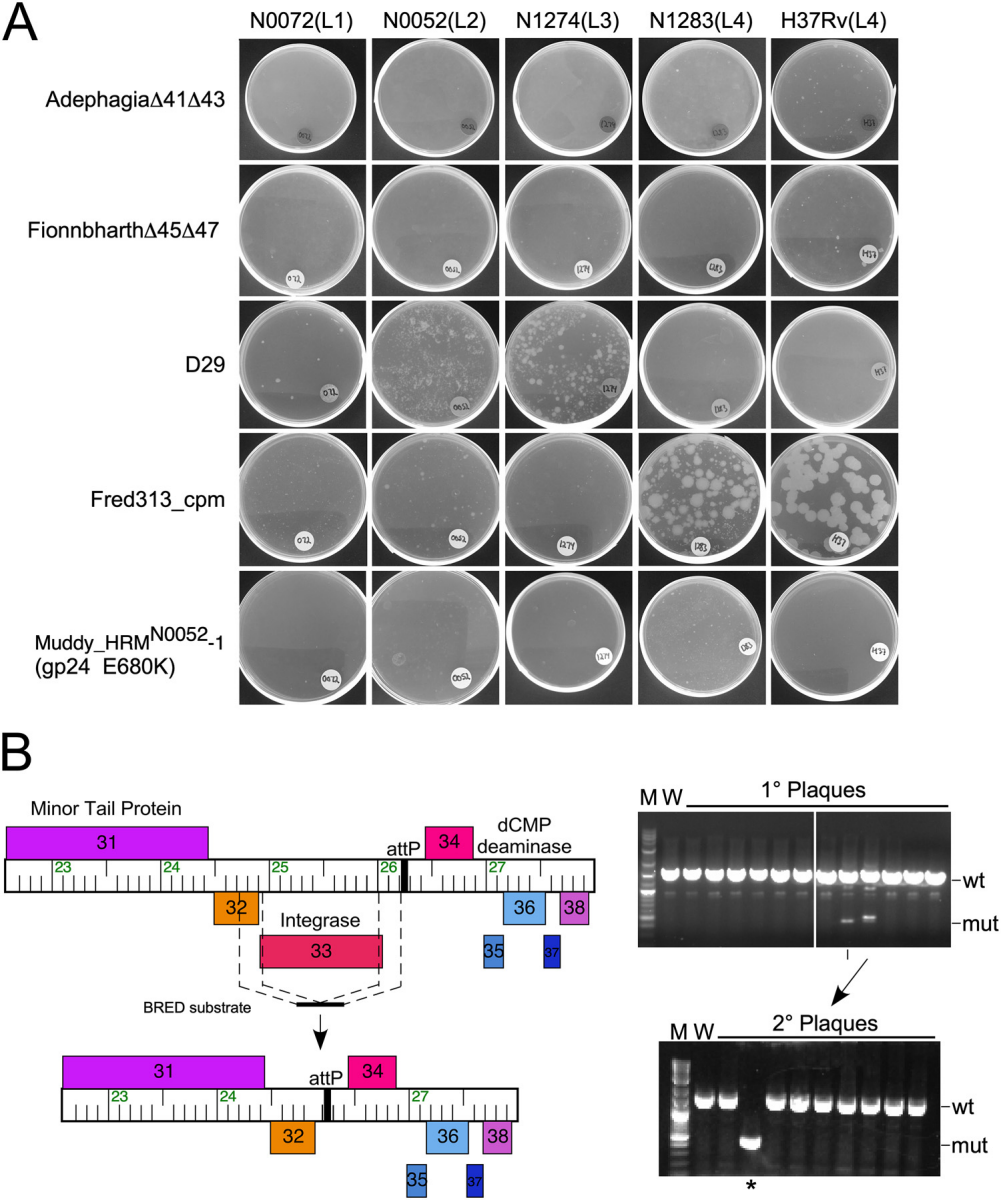
Phage resistance of M. tuberculosis strains. (A) Approximately 10^7^ CFU of each M. tuberculosis strain (as indicated above with lineage shown in parentheses) was challenged with 10^7^ to 10^8^ PFU of phage in liquid medium for 1 week and plated onto solid medium. Plates were incubated for 4 weeks. (B) Engineering of Fred313_cpm. On the left is shown a map of part of the Fred313_cpm genome with genes shown as colored boxes with the gene name within each box. Genes shown above and below the genome rule are transcribed rightward and leftward, respectively. The position of the BRED substrate is indicated, and below is the structure of the Fred313_cpmΔ*33* mutant in which the integrase gene has been removed. On the right is shown (top) PCR amplification of primary plaques recovered from BRED, all of which contain the wild-type allele (wt) and one also containing the mutant (mut) corresponding to the predicted size. After replating the indicated plaque for purification, secondary plaques were screened by PCR (bottom), one of which (asterisk) is homogenous for the desired mutation. The complete genome was sequenced to confirm the desired construction.

Little is known about mycobacteriophage receptors and the frequency or mechanisms of phage resistance. Prior studies have shown that overexpression of the M. smegmatis *mpr* (multiple-phage-resistance) locus confers resistance of M. smegmatis to phages D29 and L5 (49), and interruptions in glycopeptidolipid (GPL) synthesis confer M. smegmatis resistance to phage I3 (50). To determine the ability of M. tuberculosis to survive phage infections, ∼10^7^ CFU of each strain were challenged with phages at a multiplicity of infection (MOI) of 1 to 10 in liquid culture, incubated for 1 week, and then plated on solid medium for bacterial growth. This analysis included H37Rv and a representative strain from lineages L1 to L4, with five phages from those identified above that infect these strains efficiently (Fig. 4A).

**FIG 5:**
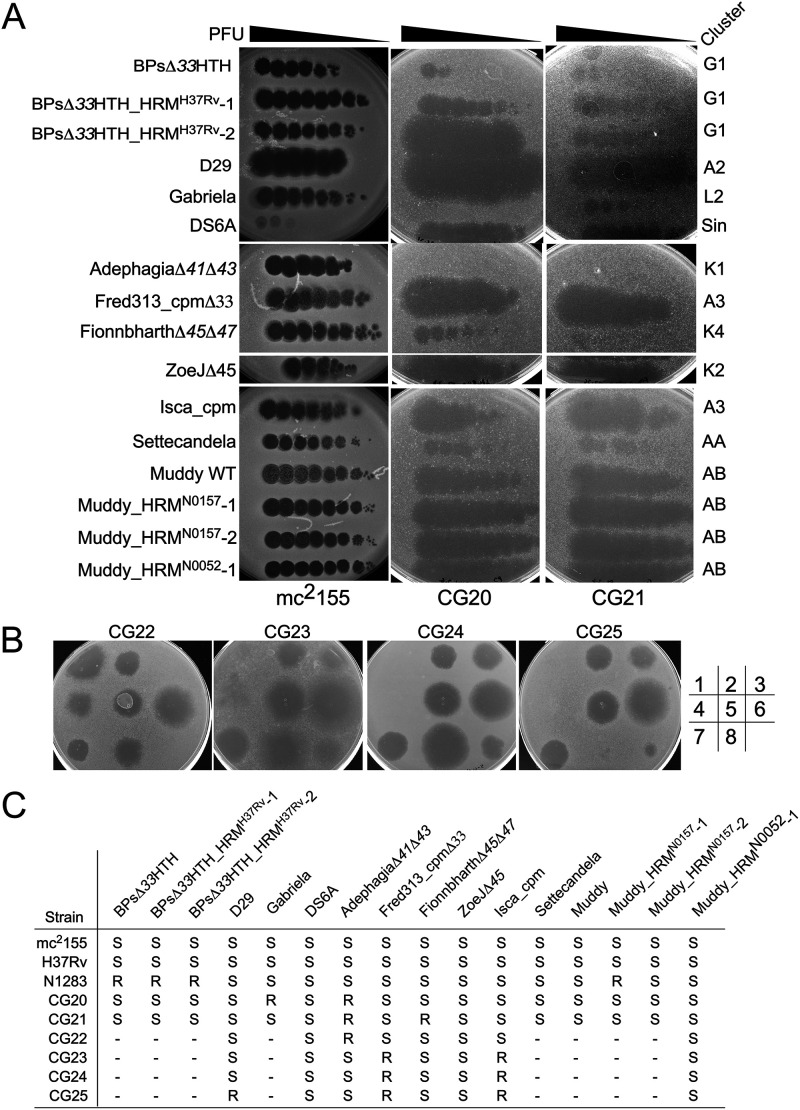
Cross resistance of phage-resistant mutants. Phage-resistant mutants CG20, CG21, CG22, CG23, CG24, and CG25 were purified and plated onto agar lawns. (A) Cross resistance was assessed by spotting phage dilutions onto strains CG20 and CG21 as shown in Fig. 1 and 2. (B) Cross resistance to other phages was determined by spotting 5 μl of single 10^−1^ dilutions (∼5 × 10^6^ to 5 × 10^7^ PFU) onto agar lawns of resistant mutants CG22, CG23, CG23, and CG25. A numbered coordinate grid (right) indicates which phage was plated as follows: 1, Fred313_cpmΔ33; 2, FionnbharthΔ45Δ47; 3, AdephagiaΔ41Δ43; 4, Isca_cpm; 5, Muddy HRM^0052^-1; 6, ZoeJΔ45; 7, DS6A; 8, D29. (C) Tabulated summary of cross-resistance observed for all resistance mutants; S, sensitive; R, resistant.

For many strain-phage combinations, the killing efficiency is impressive, and few, if any, survivors are recovered (Fig. 4A). The notable exceptions are the survivors seen on D29 infection of N0052 (L2) and N1274 (L3), and the Fred313_cpm infection of N1283 (L4) and H37Rv (L4) (Fig. 4A). We estimate that the survivor frequencies are <10^−5^ in each instance. Surviving colonies were picked wherever possible, restreaked, grown in liquid cultures and tested for resistance. Although phage Muddy_HRM^N0052^-1 (gp24 E680K) efficiently kills all of the tested strains with nearly no survivors, a few very small colonies were observed, although these could not be further propagated and retested. We were similarly unable to recover genetically stable D29-resistant mutants (colonies either did not grow or retested as being D29 susceptible). In contrast, two resistant strains to AdephagiaΔ*41*Δ*43* (from H37Rv and N1283), a Fionnbharth-resistant mutant of H37Rv, and three Fred313_cpm-resistant mutants (two in H37Rv and one in N1283) were isolated (Fig. 5A).

Sequencing of the resistant mutants and their sensitive parent strains identified mutations likely responsible for resistance to Adephagia and Fionnbharth (Table 2). The H37Rv Adephagia-resistant mutant CG20 has a single base deletion (C1939970Δ) in gene *Rv1712* (*cmk*) coding for a cytidylate kinase (51), and the frameshift (at codon 132) likely inactivates Rv1712, although it could also be polar on the downstream gene *Rv1713* coding for EngA. The H37Rv Fionnbharth-resistant mutant CG21 has a T1166874C mutation in a short, highly expressed noncoding region immediately upstream of Rv1043C, a putative serine protease. It is unclear if this region codes for a small regulatory RNA product or a small leader peptide, but it suggests an intriguing resistance mechanism. Multiple nucleotide changes were observed in the CG22 mutant and the cause of the resistant phenotype could not be readily determined. It is unclear whether these mutants indirectly alter the cell surface and prevent efficient phage adsorption, or if they influence phage metabolism after DNA injection.

Finally, sequencing of the Fred313_cpm-resistant mutants CG23, CG24, and CG25 showed that all three have complex and scrambled arrangements of Fred313_cpm DNA segments integrated at the *attB* site. At least for CG23 and CG24, we could not identify any mutations elsewhere, suggesting that these integrated prophage fragments are responsible for the resistance phenotype. The integrated phage fragments presumably lack lytic or inhibitory activity but could be associated with the resistant phenotype. At the time of this experiment, the integrase-deleted strain of Fred313_cpm had not been constructed. This is an important finding, as it strongly indicates that if lytic derivatives of temperate phages are to be used therapeutically, it would be prudent to delete not only the repressor gene, but also the integrase gene. We thus constructed the integrase-defective derivative Fred313_cpmΔ*33* using BRED engineering (52) (Fig. 4B) and this derivative was used in all other experiments reported here. Although further analysis of the numbers and types of resistance mechanisms is warranted, these observations enable examination of cross-resistance patterns, which are critical for defining compositions of phage cocktails.

### Patterns of cross-resistance to phages.

The six resistant mutants (CG20 to CG25) were propagated and tested for sensitivity to other M. tuberculosis phages (Fig. 5). In general, there are few examples of cross-resistance and they mostly occur between closely related phages (in either the same cluster or subcluster). For example, in testing CG20 and CG21 (resistant to Adephagia and Fionnbharth, respectively) for sensitivity against a panel of potentially useful phages, CG21 is resistant to Adephagia (subcluster K1) as well as Fionnbharth (subcluster K4) (Fig. 5A). However, the pattern is nonreciprocal, as CG20 remains largely sensitivity to Fionnbharth, albeit with a reduced EOP (Fig. 5A); the Adephagia-resistant mutant derived from N1283 (Table 2) also remains sensitive to Fionnbharth (Fig. 5B). All of these mutants are sensitive to ZoeJ (subcluster K2). Thus, cross-resistance within a cluster can be observed, but phages in different subclusters can have distinct sensitivities to the resistant mutants. Similarly, all three of the Fred313_cpm (subcluster A3) resistant mutants are also resistant to Isca (subcluster A3), and the N1283-derived mutant CG25 is also resistant to D29 (subcluster A2; Fig. 5B and C). In a relatively uncommon incidence of *trans*-cluster resistance, CG20 is also resistant to Gabriella (subcluster L2) (Fig. 5A). We note that all of the mutants tested are sensitive to DS6A, ZoeJΔ*45*, and Muddy_HRM^N0052^-1; Fig. 5C).

### Tuberculocidal activity of mycobacteriophages.

**FIG 6:**
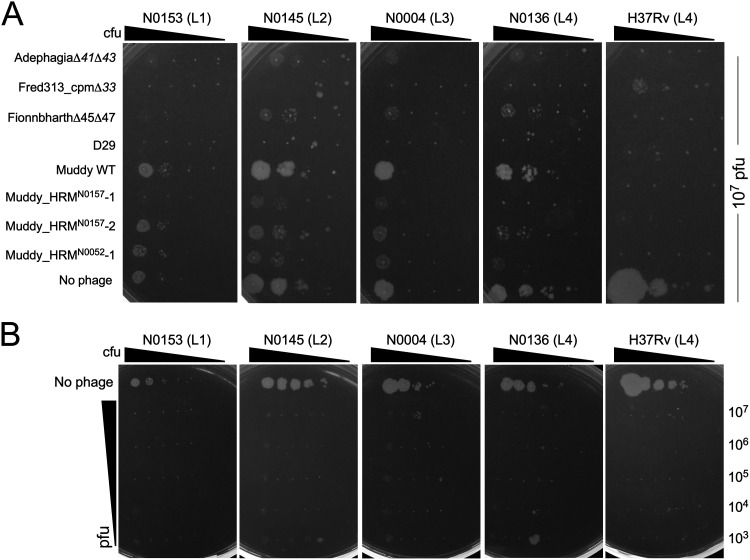
Killing efficiencies of individual phages and the five-phage cocktail for M. tuberculosis lineages. (A) A 10-fold dilution series of each of five M. tuberculosis strains (with lineages shown in parentheses) were prepared with the least dilute on the left at ∼10^7^ CFU total and incubated in liquid medium for 7 days with phages (as indicated on left) each at a total of 10^7^ PFU. Aliquots of 3 μl (∼3 × 10^4^ CFU at 10^−1^ dilution) were then plated onto solid medium and incubated for 4 weeks at 37°C. (B) Dilutions of M. tuberculosis strains were prepared as in panel A and incubated in liquid culture with a five-phage cocktail containing equal amounts of AdephagiaΔ*41*Δ*43*, Fred313_cpmΔ*33*, FionnbharthΔ*45*Δ*47*, Muddy_HRM^N0157^-1 (gp24 G487W), and D29. The top rows contain a total of 10^7^ PFU, and below are shown 10-fold serial dilutions of the phage input.

Using the information gained from the cross-resistance studies, we examined the tuberculocidal activity of both individual phages and a cocktail of phages. Cultures of representative M. tuberculosis strains were grown until visibly turbid (optical density [OD] of ∼0.1), serially diluted, and incubated with individual phages in liquid medium for 96 h. These were then plated onto solid medium for growth of survivors (Fig. 6A). Most of the individual phages killed the strains quite efficiently, even with a relatively modest input concentration of phage (10^7^ PFU, although killing was often incomplete at the highest input bacterial concentration). For strain N0004, growth was only observed for the least dilute sample of the control, and the killing efficiency is less clear. Muddy WT did not kill any strain well, and the Muddy host range mutants did not efficiently kill N0145 (Fig. 6A).

We then tested the tuberculocidal activity of a cocktail of five phages, AdephagiaΔ*41*Δ*43*, D29, FionnbharthΔ*45*Δ*47*, Fred313_cpmΔ*33*, and Muddy_HRM^N0^**^157^**-2, the phages used above to test for resistance (but substituting Fred313_cpmΔ*33* for Fred313_cpm; Fig. 4). This combination of phages maximizes the proportion of strains that are infected and killed by more than one phage and thus minimizes the risks of resistance emerging (Table 3). M. tuberculosis H37Rv and representative strains of lineages L1 to L4 (N0153, N0145, N0004, and N0136) were incubated with the phage cocktail at a range of 10^7^ to10^3^ total PFU for 7 days and then plated on solid medium for bacterial growth (Fig. 6B). Very strong killing and little or no survival at any concentration of phage or bacteria was observed, with the exception of the lowest phage concentration with strain N0136 (Fig. 6B). We also tested a similar cocktail (substituting Muddy HRM^N0157^-1 for Muddy HRM^N0052^-1) with strains N0052 (L4), N0054 (L4), and N1283 (L4) with similar results, and as few as 10^5^ PFU input phage gave substantial killing within 24 h (Fig. S1). Although the cocktail likely could be further enhanced with other phage combinations, the tuberculolcidal activity is impressive and is strongly encouraging for therapeutic use.

### Phage and antibiotic combinations.

**FIG 7:**
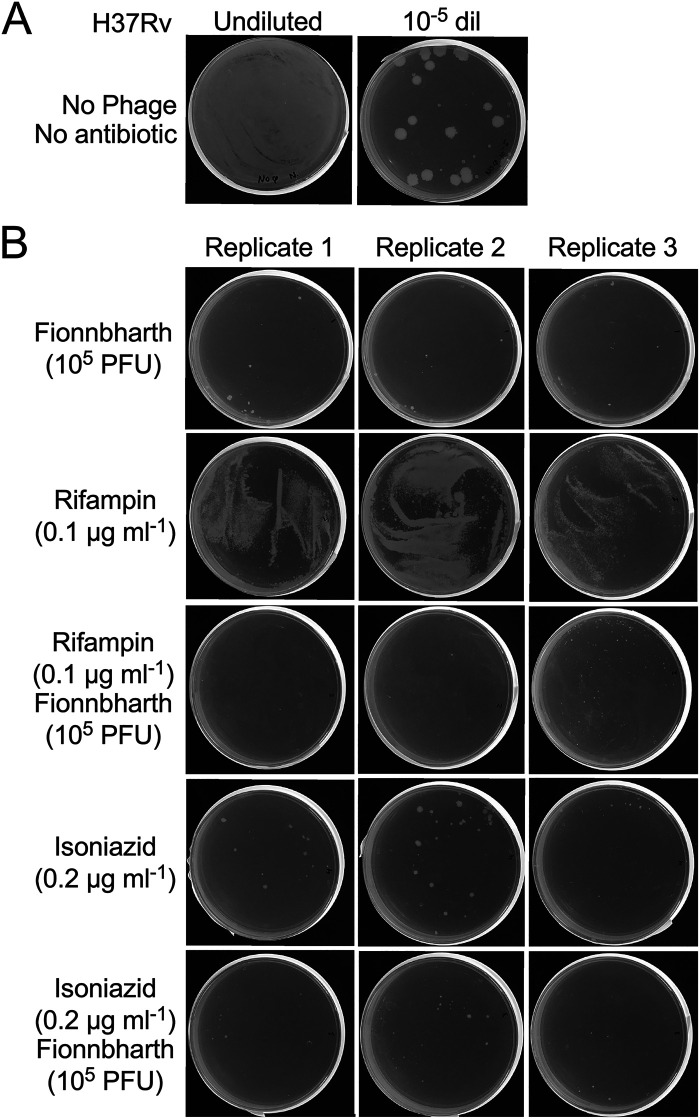
Phage and antibiotic interactions. (A) Controls of input M. tuberculosis H37Rv in the experiment. The left and right panels show plating of 100 μl of an undiluted culture of M. tuberculosis H37Rv and a 10^−5^ dilution, respectively. (B) Aliquots (100 μl) of an undiluted culture of M. tuberculosis H37Rv were plated directly onto solid medium containing either rifampin or isoniazid at the final concentrations indicated, or onto plates on which 10^9^ PFU of Fionnbharth had been added and spread over the agar surface. Plates were incubated for 4 weeks.

Potential therapeutic use of phages for tuberculosis is likely to be accompanied by antibiotic treatment. It is therefore important that antibiotics, especially the commonly used isoniazid and rifampin, do not antagonize phage growth and killing. To test this, H37Rv was plated on solid medium with sub-MICs of either isoniazid or rifampin alone, or each of the drugs together with 10^5^ PFU FionnbharthΔ*45*Δ*47* (Fig. 7). In all antibiotic-phage combinations, similar levels of killing were observed, and there was no evidence of antagonism, reflecting what has been reported in M. smegmatis (53). Under these conditions, it is not possible to draw strong conclusions about synergistic or additive effects of antibiotic and phage, but we note that the few surviving colonies with the FionnbharthΔ*45*Δ*47* challenge are not observed when rifampin is included, suggesting the effects are at least additive. Similarly, fewer surviving colonies are recovered after challenge with both isoniazid and FionnbharthΔ*45*Δ*47* than with either alone. In this instance, the lack of antagonism between phage and antibiotics is particularly encouraging, as it suggests that adjunctive phage therapy with ongoing antibiotic treatment is unlikely to cause a poor outcome due to antibiotic interference.

**FIG 8:**
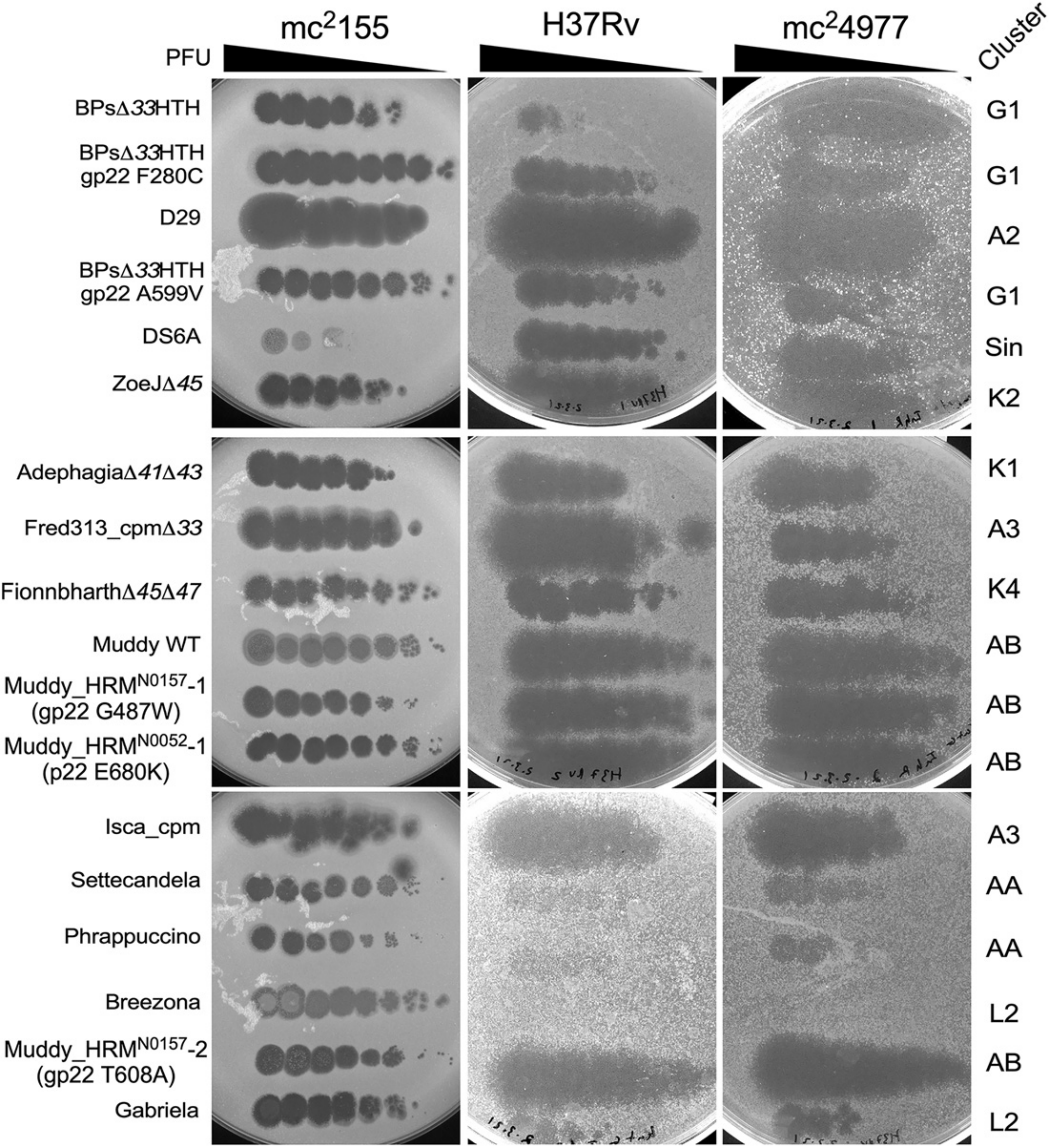
Phage infection of M. tuberculosis mc^2^4977. Ten-fold serial dilutions of phages as shown on the left were spotted onto lawns of M. smegmatis mc^2^155, M. tuberculosis H37Rv, and M. tuberculosis mc^2^4977, which is isoniazid resistant due to deletion of the *katG* gene.

It is also important that therapeutically useful phages are able to infect antibiotic-resistant as well as antibiotic-sensitive strains. Because isoniazid inhibits cell wall mycolic acid synthesis and isoniazid resistance is common via loss of KatG function, we compared the phage susceptibility of a *katG* (del 371g) isoniazid-resistant strain (mc^2^4977) with H37Rv (Fig. 8). Only small differences in phage susceptibility were observed, including a slight difference in the infection with Fred313_cpmΔ*33* (Fig. 8). Interestingly, the parent BPsΔ33HTH phage, which does not infect H37Rv well, appears to infect mc^2^4977 quite efficiently (Fig. 8). Because drug-resistant M. tuberculosis strains accumulate individual target gene mutations rather than defects in single-locus drug exporters, it is relatively unlikely that other drug-resistant strains will have markedly different phage infection profiles.

### Phage coevolution to overcome resistance.

**FIG 9:**
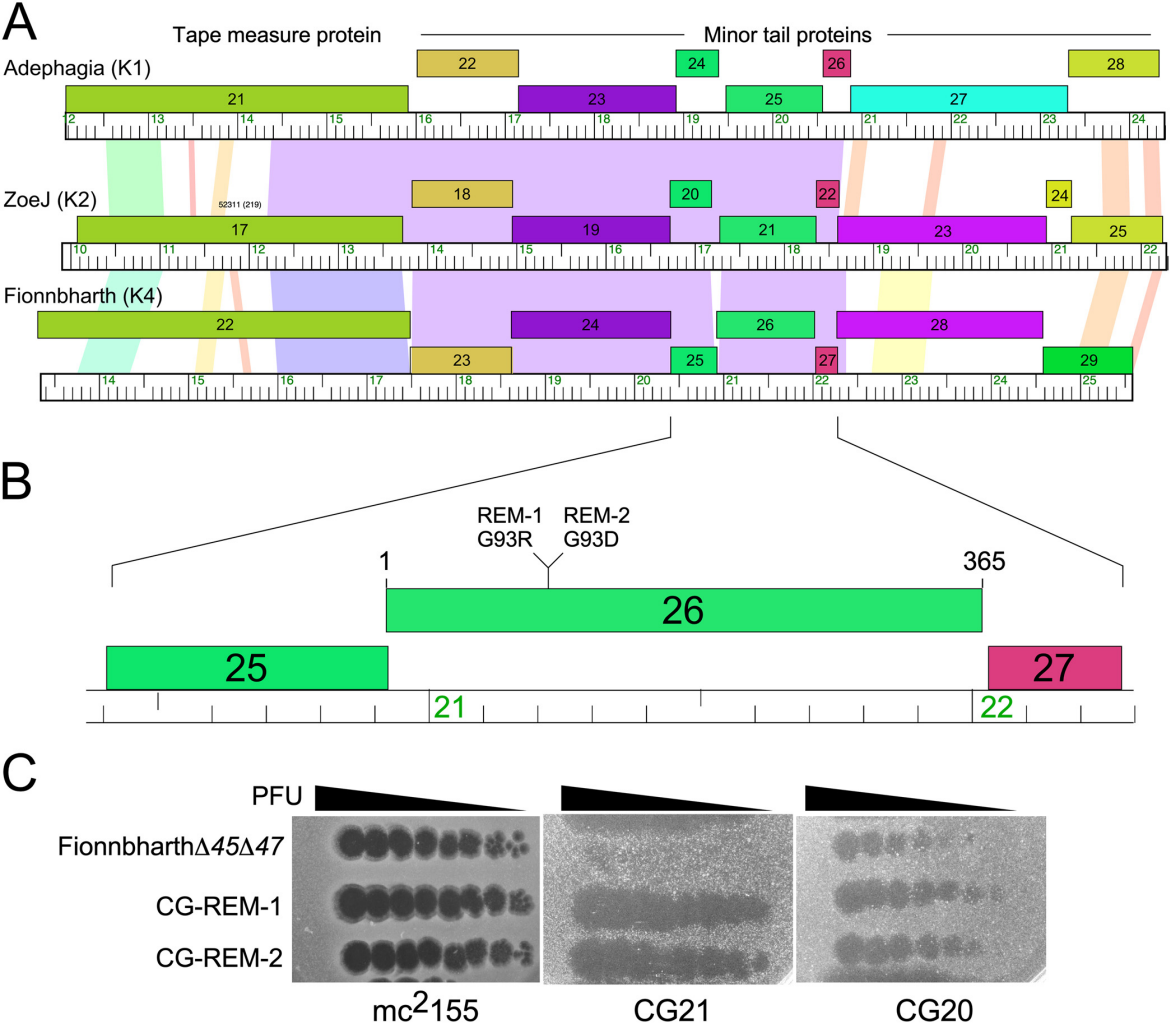
Fionnbharth resistance escape mutants. (A) Alignment of the tail gene segments of Adephagia, ZoeJ, and Fionnbharth (subclusters K1, K2, and K4, respectively) genomes shows the location of Fionnbharth gene *26*, coding for a putative phage tail protein. Genes are shown as colored boxes with gene numbers within the boxes, with coloring reflecting similar phamilies of protein sequences. Spectrum-colored shading between the genomes reflects nucleotide sequence similarity, with violet being the most similar, and red the least similar above a threshold E value of 10^−4^ (64). (B) An expanded view of Fionnbharth gene *26* showing the locations of two mutations conferring substitutions (G93R and G93D) in the resistance escape mutants REM-1 and REM-2, respectively. (C) Phage infections of Fionnbharth and CG-REM-1 and CG-REM2 mutants on lawns of M. smegmatis mc^2^155, CG20, and CG21.

Because phage resistance is a concern in any clinical phage application, we determined if phage derivatives can be isolated that escape resistance (Fig. 9). When plating FionnbharthΔ*45*Δ*47* on CG21 (a Fionnbharth-resistant mutant of M. tuberculosis H37Rv), we observed two healthy growing plaques (from ∼10^8^ PFU input phage). These were purified, retested, and shown to be escape mutants (CG-REM-1 and CG-REM-2) that infect the resistant strain as efficiently as the parent H37Rv strain (Fig. 9C). Whole-genome sequencing showed that both mutants have nonsynonymous base changes (G21203A and G21202C in CG-REM-1 and CG-REM-2, respectively) conferring G93R and G93D substitutions in the minor tail protein, gp26 (Fig. 9B).The minor tail protein gp26 is highly conserved in cluster K phages, including Adephagia gp25 and ZoeJ gp21 (Fig. 9A), and there are related proteins in many other mycobacteriophages. Interestingly, although CG21 is resistant to both Fionnbharth and Adephagia, it remains sensitive to ZoeJ (Fig. 5A). The isolation of resistant escape mutants presents a potentially powerful response to the emergence of phage resistance.

## DISCUSSION

There is considerable clinical potential for using mycobacteriophages in tuberculosis control, as diagnostic reporter phages (54–56), for prophylactic interruption of TB transmission (38, 39), or for therapeutic treatment of infections (38). All of these are advanced by identification of particular phage candidates, elucidating mechanisms of resistance and cross-resistance, and determining variations in infection for different strains and genetic lineages. The potential for therapeutic use of phages for controlling TB infections directly is unclear because of the complexities of the infections in which the pathogen lives intracellularly in macrophages, and within inaccessible granulomas. Nonetheless, at late stages of infection there are often substantial numbers of extracellular bacteria that should be phage-accessible, and the successful therapy of an M. abscessus infection provides substantial encouragement (33). Nonetheless, the phage infection profiles in an infected person may not directly correlate with the *in vitro* susceptibilities reported here. However, resolving this question will likely require clinical trials, compassionate use interventions, or evaluation in nonhuman primates. In addition, future studies will be needed to more fully explore phage-antibiotic interactions with an expanded repertoire of phages, drugs, and M. tuberculosis strains.

One potential advantage of phage control of M. tuberculosis is that there is relatively little variation among clinical isolates in terms of phage susceptibility compared to other pathogens such as M. abscessus (36). The early phage typing studies showed that some phages infect a broad range of M. tuberculosis isolates, although other phages discriminate between some strains. Here, we have expanded this in the context of genomically defined phages and broadened the available phages through a combination of engineering and genetics. These studies suggest that a cocktail containing as few as five phages, as shown here, might be suitable for use in clinical trials for phage efficacy and safety. Moreover, the phage cocktail could be deployed with minimal concerns of failure due to resistance, and without the need to prescreen patient isolates for phage susceptibility, a process that would be technically and logistically challenging with such slow-growing bacteria. Having confidence in the ability of a five-phage cocktail to kill a very high proportion of strains offers a substantial advantage over almost every other pathogen for which phage therapy is contemplated.

The five-phage cocktail tested here is likely to undergo further refinement prior to clinical evaluation. For example, ZoeJΔ*45* could substitute for Adephagia, as it showed no cross-resistance to Fionnbharth, and one of the FionnbharthΔ*45*Δ*47* resistance escape mutants (e.g., CG21) could replace FionnbharthΔ*45*Δ*47* as a means of further reducing resistance. A case can also be made for inclusion of DS6A, which broadly infects and kills the tested strains. Two potential caveats are that DS6A processes an integration cassette (43), which should be removed, and that it needs to be amplified and propagated on a slow-growing MTBC strain, which is time-consuming and challenging at large scale. There is also potential for additional phages to be developed, including lytic variants of Gabriela and Settecandela, although in general these cluster AA phages did not perform as well as others. It is surprising that the BPsΔ*33*HTH_HRM mutants that infect H37Rv do not infect other M. tuberculosis strains, but it may be possible to isolate new host range mutants that expand the utility of BPs derivatives.

Although the phages and the cocktail tested here killed most of the tested strains, the exception is lineage 6, for which one of the tested strains was susceptible (N0091) but not the other (N1202) (Table 3). However, L6 strains are found in limited geographical regions and represent only a small minority of all tuberculosis infections (23); however, early clinical trials may need to avoid the regions where L6 strains are prevalent. There are additional lineages we have not yet tested, including L7, L8, and L9, although L7 is also rare and is restricted to Ethiopia, and both L8 and L9 have been reported from very few individual patients (25, 29). It would also be helpful to examine a much broader set of clinical isolates and more drug-resistant strains, especially those in lineages L2, and L4, which are more diverse, more virulent, and more likely to become drug resistant (30). Nonetheless, the broad coverage provided by these phages, especially among the diverse L2 and L4 strains, encourages us to consider it unlikely there will be large swaths of M. tuberculosis strains that that are not infected and killed by at least a subset of the cocktail phages.

Of the phages described here, only Muddy is a naturally lytic phage. All of the others are either naturally occurring or engineered lytic derivatives of temperate parent phages; all are siphoviral. Thus, the available phage “space” available for tuberculosis therapy is quite distinct from many other bacterial pathogens, for which lytic myoviruses and podoviruses have been widely used. This does appear to be an impediment, and engineering strategies can be used to convert the temperate phages into lytic phages through removal of the repressor gene. However, our finding that survivors of a Fred313_cpm challenge carry integrated phage genome segments suggests it is advisable to also remove the integrase genes. Fortunately, recombineering tools applied in the BRED and newer CRISPY-BRED methods provides simple and effective ways of doing so (52, 57).

With the identification of a set of phages that efficiently infect and kill a broad range of M. tuberculosis strains with seemingly low resistance frequencies, infrequent cross-resistance, and that work together with antibiotics and infect antibiotic-resistant strains, there are now few impediments to clinical evaluation of bacteriophages for relief of tuberculosis. Whether such therapy might be broadly applicable or restricted to a narrow spectrum of disease states is not clear, but with the excellent safety profile of phages in humans (33, 58), these questions now can be addressed.

## MATERIALS AND METHODS

### Bacterial strains and media.

M. smegmatis mc^2^155 is a laboratory stock strain and was grown as previously described (21). M. tuberculosis strains were obtained from Sebastien Gagneux Swiss Tropical and Public Health Institute. Liquid cultures were grown by inoculating isolated colonies in 10 ml Middlebrook 7H9 media with oleic albumin dextrose catalase (OADC) (Becton, Dickinson) and 0.05% Tween 80 until visibly dispersed (10 days to 3 weeks) at 37°C with shaking. Lineage 5 and 6 strains were further supplemented with 40 mM sodium pyruvate (Sigma). Strains were grown on solid Middlebrook 7H11 agar (Difco, Remel) supplemented with OADC and 1 mM CaCl_2_ for 2 to 6 weeks at 37°C .

### Phage susceptibility assays.

Phage lysates were 10-fold serially diluted and 3 μl were spotted onto top agar overlays containing 0.5 to 1 ml of M. smegmatis mc^2^155 or an M. tuberculosis strain using Middlebrook 7H11 with 0.7% agar for M. tuberculosis and Middlebrook 7H10 with 0.35% agar for M. smegmatis. Plates were incubated at 37°C for 24 to 48 h for M. smegmatis or 2 to 8 weeks for M. tuberculosis, until visible lawns were obtained. Plates were photographed and analyzed for plaque formation.

### PCR screening of Muddy host range expansion mutants.

Lysates were made from plaques forming on M. tuberculosis strains. Lysates on M. smegmatis were amplified under BSL3 conditions and were filtered twice using 0.2-μm filters. Aliquots of lysates (1 ml) were serially diluted and plated onto agar lawns for isolated plaques. Isolated plaques (*n* = 8 to16) were picked using a 0.2 to 10 μl micropipette tip into 50 μl of phage buffer (21) in 0.2-ml PCR strip tubes. An aliquot of 5 μl containing phage particles picked from agar was used as the template for PCR utilizing Muddy gp24-specific primers (Table S2) along with Q5 master mix (New England BioLabs) following PCR according to the manufacturer’s enzyme conditions. Amplicons were verified by gel electrophoresis and were sequenced (Genewiz).

### Phage engineering.

Fred313_cpmΔ*33* was constructed using bacteriophage recombineering of electroporated DNA (BRED) as described previously (34, 52) using a 500-bp gBlock substrate containing 250 bp of homology upstream and downstream of gene *33*. Approximately 400 ng of substrate and 250 ng of Fred313_cpm DNA were electroporated into competent recombineering M. smegmatis mc^2^155 cells (59) induced with acetamide. Primary and secondary plaques were screened using PCR with flanking primers yielding either a 1,634-bp or 536-bp product wild-type and mutant alleles, respectively. A homogenous mutant was purified, amplified, and sequenced. All oligonucleotides are provided in Table S2.

### Individual phage killing assay.

To assess killing of individual phages at 10^7^ PFU, phage titers were normalized to 1 × 10^9^ PFU per milliliter (PFU/ml). In a 96-well plate (Falcon), 20 μl of each phage (one per row) was added to a total volume of 200 μl consisting of Middlebrook 7H9 supplemented with OADC and 1 mM CaCl_2,_ and the bacterial strain, grown until visibly dispersed (OD_600_ of ≥0.1) and 10-fold serially diluted to 10^−1^ to 10^−4^. The bottom row of each 96-well plate contained bacteria and no phage. To assess killing of 10^4^ PFU, the phage lysate was normalized to 10^5^ PFU and then the same procedure was followed as detailed above. The plates were sealed and incubated without shaking at 37°C for 96 h. Each well was mixed by pipetting and then 3 μl was spotted onto Middlebrook 7H11 plates containing 1 mM CaCl_2_ and OADC and the plates incubated for 3 weeks at 37°C before imaging.

### Cocktail killing assay.

Phage titers were normalized to 1 × 10^8^ PFU/ml and 20 μl of each phage were combined into a cocktail. Liquid bacterial cultures were grown and aliquoted into 96-well plates as described above; the cocktail was serially diluted such that each row contained from 10^7^ to 10^3^ PFU total phage. Approximately 20 μl of serially diluted M. tuberculosis (∼5 × 10^8^ CFU/ml) from undiluted to a 10^−4^ dilution was added to each plate column. Plates were sealed and incubated standing at 37°C. At 24, 48, and 96 h and 1 week of time, the 96-well plates were centrifuged at 3,500 rpm for 2 min to remove condensation from the sealing film using a bio-liner swing bucket rotor (Thermo). Cultures were resuspended using a multichannel pipet and 3 μl aliquots were spotted onto Middlebrook 7H11 plates supplemented with OADC and 1 mM CaCl_2_ and incubated for 3 to 4 weeks at 37°C.

### Isolation of phage-resistant mutants.

Approximately 100 μl of bacterial cultures at OD of ∼0.1 to 0.2 was added to tubes containing 1 ml of 7H9 supplemented with OADC and 1 mM CaCl_2_ and 1 × 10^7^ to 1 × 10^8^ PFU of phage. After incubation with shaking (200 rpm) at 37°C for 1 week, cells were pelleted at 5,000 × *g* for 10 min, resuspended in 100 μl 7H9 OADC, and spread onto 7H11 plates containing OADC. Plates were incubated for 4 to 8 weeks and surviving colonies restreaked onto 7H11 OADC plates. Colonies that grew without evidence of lysis were inoculated into liquid culture and tested for phage sensitivity.

### Isolation of phage resistance escape mutants.

Approximately 3 μl of phage lysates (10^9^ to 10^11^ PFU/ml) were spotted onto lawns of phage-resistant mutants and individual plaques picked and replated on the resistant mutant and M. smegmatis mc^2^155 to determine the EOP. Plaques were picked from the M. smegmatis mc^2^155 lawn and replated on the M. tuberculosis resistant mutant. True-breeding escape mutants were amplified and sequenced.

### Phage and antibiotic interactions.

Middlebrook 7H11 plates were prepared to contain rifampin (Sigma; 0.1 μg/ml) or 0.2 μg/ml isoniazid (Sigma; 0.2 μg/ml). Phage lysate diluted to 10^5^ PFU in 0.1 ml was spread onto 7H11 plates with or without antibiotics and allowed to dry in a laminar flow biosafety cabinet; 0.1 ml of an M. tuberculosis H37Rv culture was then spread into plates and incubated for 6 weeks at 37°C.

### DNA isolation, sequencing, and variant detection.

Extraction of M. tuberculosis and phage DNAs was as described previously (60, 61). Bacterial and phage genomes were sequenced using Illumina technology as described previously (36, 62), and details of the sequenced strains are shown in Table S3. Sequence reads of mutants were aligned to parent sequences in CLC Genomics Workbench 11 (Qiagen), and variants were detected using CLC’s Basic Variant Detection module and confirmed in Consed version 29 (63).

### Data availability.

GenBank accession numbers for *M. tuberculosis* phage-resistant isolates CG20 to CG25 are available in Table S3.

10.1128/mBio.00973-21.1 TABLE S1 Major type phage (MTPH) reported previously for typing M. tuberculosis isolates. Download Table S1, PDF file, 0.03 MB. Copyright © 2021 Guerrero-Bustamante et al. 2021 Guerrero-Bustamante et al. https://creativecommons.org/licenses/by/4.0/ This content is distributed under the terms of the Creative Commons Attribution 4.0 International license.

10.1128/mBio.00973-21.2 TABLE S2 Oligonucleotides used in this study. Download Table S2, PDF file, 0.05 MB. Copyright © 2021 Guerrero-Bustamante et al. 2021 Guerrero-Bustamante et al. https://creativecommons.org/licenses/by/4.0/ This content is distributed under the terms of the Creative Commons Attribution 4.0 International license.

10.1128/mBio.00973-21.3 TABLE S3 Sequencing details of Mycobacterium strains. Download Table S3, PDF file, 0.02 MB. Copyright © 2021 Guerrero-Bustamante et al. 2021 Guerrero-Bustamante et al. https://creativecommons.org/licenses/by/4.0/ This content is distributed under the terms of the Creative Commons Attribution 4.0 International license.

10.1128/mBio.00973-21.4 FIG S1 Killing efficiencies of individual phages and the five-phage cocktail for M. tuberculosis lineages. (A) Dilutions of M. tuberculosis strains were prepared as in Fig. 6B and incubated in liquid culture with a five-phage cocktail containing equal amounts of AdephagiaΔ*41*Δ*43*, Fred313_cpmΔ*33*, FionnbharthΔ*45*Δ*47*, Muddy_HRM^N0157^-1, and D29. The top rows contain a total of 10^7^ PFU, and below are shown 10-fold serial dilutions of the phage input. Cocktail was spotted onto agar plates at 24 h, 48h, 96 h, and 1 week at 37°C as indicated. (B) The same experiment as in panel A, but using a cocktail containing AdephagiaΔ*41*Δ*43*, Fred313_cpmΔ*33*, FionnbharthΔ*45*Δ*47*, Muddy_HRM^N0052^-1, and D29. Download FIG S1, PDF file, 1.5 MB. Copyright © 2021 Guerrero-Bustamante et al. 2021 Guerrero-Bustamante et al. https://creativecommons.org/licenses/by/4.0/ This content is distributed under the terms of the Creative Commons Attribution 4.0 International license.
