# Supplementary material for: Toward a Phage Cocktail for Tuberculosis: Susceptibility and Tuberculocidal Action of Mycobacteriophages against Diverse Mycobacterium tuberculosis Strains
Source: mBio. 2021 May 20;12(3):e00973-21. doi: 10.1128/mBio.00973-21 (PMC8263002; doi:10.1128/mBio.00973-21)
Supplement: TABLE S1 [file mbio.00973-21-st001.pdf]

Table S1. Major Type Phage (MTPH) reported previously for typing *M. tuberculosis* isolates

MTPH[1]	Phage	Propagation host[1]	Cluster	%GC	Accession #	Reference
1	AG1	*M. kansasii* At7	N/A	N/A	N/A[2]	(1)
2	DS6A	*M. tuberculosis* H37Rv	Sin	68.40	JN698994	(2)
3	GS4E	*M. tuberculosis* H37Rv	A2	63.4	AP018479	(3)
4	BK1	*M. smegmatis* ATCC 607	A1	63.4	AP018477	(3)
5	BG1	*M. intracellulare* P-17	N/A	N/A	N/A	(4)
6	D34	Uncharacterized *M. sp.* F130	N/A	N/A	N/A	(5)
7	DNA III	*M. tuberculosis* H37Rv	G1	67	KC787108.1	(6)
8	X20	*M. tuberculosis* H37Rv	N/A	N/A	N/A	(7)
9	PH	*M. tuberculosis* H37Rv	N/A	N/A	N/A	(8)
10	Clark	*M. smegmatis* ATCC 607	G1	66	KC787112.1	(6)
11	Sedge	*M. smegmatis* ATCC 607	G1	66	KC787111.1	(6)
12	Legendre	*M. smegmatis* ATCC 607	G1	67	KC787109.1	(6)

[1]MTPH and propagation host are as reported previously (9, 10)
[2]N/A, Not Available

References

1. Redmond WB, Cater JC, Ward DM. 1963. Spotting method of phage typing mycobacteria. Typing mycobacteria with routine test dilutions. Am Rev Respir Dis 87:257-63.
2. Pope WH, Jacobs-Sera D, Russell DA, Peebles CL, Al-Atrache Z, Alcoser TA, Alexander LM, Alfano MB, Alford ST, Amy NE, Anderson MD, Anderson AG, Ang AA, Ares M, Jr., Barber AJ, Barker LP, Barrett JM, Barshop WD, Bauerle CM, Bayles IM, Belfield KL, Best AA, Borjon A, Jr., Bowman CA, Boyer CA, Bradley KW, Bradley VA, Broadway LN, Budwal K, Busby KN, Campbell IW, Campbell AM, Carey A, Caruso SM, Chew RD, Cockburn CL, Cohen LB, Corajod JM, Cresawn SG, Davis KR, Deng L, Denver DR, Dixon BR, Ekram S, Elgin SC, Engelsen AE, English BE, Erb ML, Estrada C, Filliger LZ, et al. 2011. Expanding the diversity of mycobacteriophages: insights into genome architecture and evolution. PLoS One 6:e16329.
3. Uchiyama J, Mizukami K, Yahara K, Kato S-I, Murakami H, Nasukawa T, Ohara N, Ogawa M, Yamazaki T, Matsuzaki S, Sakaguchi M. 2018. Genome Sequences of 12 Mycobacteriophages Recovered from Archival Stocks in Japan. Genome announcements 6:e00472-18.
4. Jones WD, Jr., Greenberg J. 1978. Modification of methods used in bacteriophage typing of Mycobacterium tuberculosis isolates. J Clin Microbiol 7:467-9.
5. Rado TA, Bates JH, Fitzhugh JK. 1976. Evidence for host-dependent modification and restriction of bacteriophage DNA in Mycobacterium tuberculosis. J Gen Virol 30:91-7.
6. Mankiewicz E. 1972. Bacteriophage Types of Mycobacteria. Canadian Journal of Public Health / Revue Canadienne de Sante'e Publique 63:342-354.
7. Mankiewicz E, Liivak M. 1975. Phage types of mycobacterium tuberculosis in cultures isolated from Eskimo patients. Am Rev Respir Dis 111:307-12.
8. Sushida K, Hirano N. 1972. Isolation from media of two bacteriophages, protamylase-H37Rv (PH) active against Mycobacterium tuberculosis. Am Rev Respir Dis 106:269-71.
9. Rado TA, Bates JH, Engel HW, Mankiewicz E, Murohashi T, Mizuguchi Y, Sula L. 1975. World Health Organization studies on bacteriophage typing of mycobacteria. Subdivision of the species Mycobacterium tuberculosis. Am Rev Respir Dis 111:459-68.
10. Garcia-Rodriguez JA, Gomez-Garcia AC, Aguero J. 1986. Preliminary studies on bacteriophage typing of Mycobacterium tuberculosis strains isolated in Salamanca (Spain). Eur J Epidemiol 2:178-81.
