# Supplementary material for: Toward a Phage Cocktail for Tuberculosis: Susceptibility and Tuberculocidal Action of Mycobacteriophages against Diverse Mycobacterium tuberculosis Strains
Source: mBio. 2021 May 20;12(3):e00973-21. doi: 10.1128/mBio.00973-21 (PMC8263002; doi:10.1128/mBio.00973-21)
Supplement: TABLE S2 [file mbio.00973-21-st002.pdf]

Table S2. Oligonucleotides used in this study

Primer name	Primer sequence	Use
Muddy_gp24_Fwd	CGCTGATGCTACAAGGTTTTAC	to amplify Muddy gp24
Muddy_gp24_Rev	GCCGTTGACATACCAGACG	to amplify Muddy gp24
Fred313_cpm_33gBlock	GGCGAAAACACCTCCTGACCTGCGGAGCGGGCGACGG<br>GAATCGAACCCGCGTAGCTAGTTTGGAAGAAAGGGTG<br>TCGTCTGGAGCTGTTCCAGCAGGTCAGACTAGATTTTT<br>ACCCCCTCCCTACTGCAACGCTGAAGTTGAAAGAAATT<br>GCAGGTCGCGGCAGCGTGTTGAGTCTCGGGAGTTGCA<br>ATAGAGTTGCAAATCGGTACCCTCTCTGTCGGGAGAAA<br>GGGGACCTAGTTGGCACCATCACGAAAGGCCAGGTCC<br>TGAAGGAAGGAGAACAATGCACAAACTCGCTCTCACTC<br>TGACGGCAGCAGCGGTCCTGCTGGCCGGGTGCAGCCA<br>GGAAGCTCCCTCGGCAGCTCCAACCGCTCCAGCCGCCA<br>AGGAAGAGGCGAAGCGGGGAACCGTGGTCTTCGAGAT<br>CGGTGGCAACTACAGCTACGCGACCTACGACGACAACT<br>TCGAGAACGGCATCGAGTACCCGCCTGGCGTCACCCGG<br>ATCGAGTTGCAC	gBlock
Fred313_cpm_33gBlockF	GGCGAAAACACCTCCTGACCT	to amplify gBlock
Fred313_cpm_33gBlockR	GTGCAACTCGATCCGGGTGAC	to amplify gBlock
Fred313_cpm_33checkF	TGCAGAGGGTCTGCAACTCT	to check candidates
