# Supplementary material for: Toward a Phage Cocktail for Tuberculosis: Susceptibility and Tuberculocidal Action of Mycobacteriophages against Diverse Mycobacterium tuberculosis Strains
Source: mBio. 2021 May 20;12(3):e00973-21. doi: 10.1128/mBio.00973-21 (PMC8263002; doi:10.1128/mBio.00973-21)
Supplement: TABLE S3 [file mbio.00973-21-st003.pdf]

Table S3. Sequencing details of *Mycobacterium tuberculosis* parent and mutant strains

Strain[1]	Length (bp)[2]	Contigs[3]	Coverage[4]	Status[5]	GenBank Accession
H37Rv_CG	4415999	1	132	Complete	CP072765
N1283	4365552	207	140	WGS	JAGKIM000000000
CG20	4415998	1	133	Complete	CP072764
CG21	4415999	1	104	Complete	CP072763
CG22	4363707	165	95	WGS	JAGKIN000000000
CG23	4432513	1	135	Complete	CP072762
CG24	4459449	1	111	Complete	CP072761
CG25	4398881	170	109	WGS	JAGKIO000000000

[1]Strains are given a CGXX designation.
[2]For complete genomes, the length is the precise length of the bacterial chromosome. For WGS genomes, length is the sum of the lengths of all assembled contigs and therefore may be smaller or larger than the true genome size.
[3]For WGS genomes, the number of contigs obtained through sequencing and assembly with Unicycler.
[4]Genome coverage obtained through WGS sequencing for each strain.
[5]Genome sequencing with Illumina whole genome sequencing or Illumina and Nanopore to completion.
.
