# Supplementary figures and images for: Toward a Phage Cocktail for Tuberculosis: Susceptibility and Tuberculocidal Action of Mycobacteriophages against Diverse Mycobacterium tuberculosis Strains
Source: mBio. 2021 May 20;12(3):e00973-21. doi: 10.1128/mBio.00973-21 (PMC8263002; doi:10.1128/mBio.00973-21)

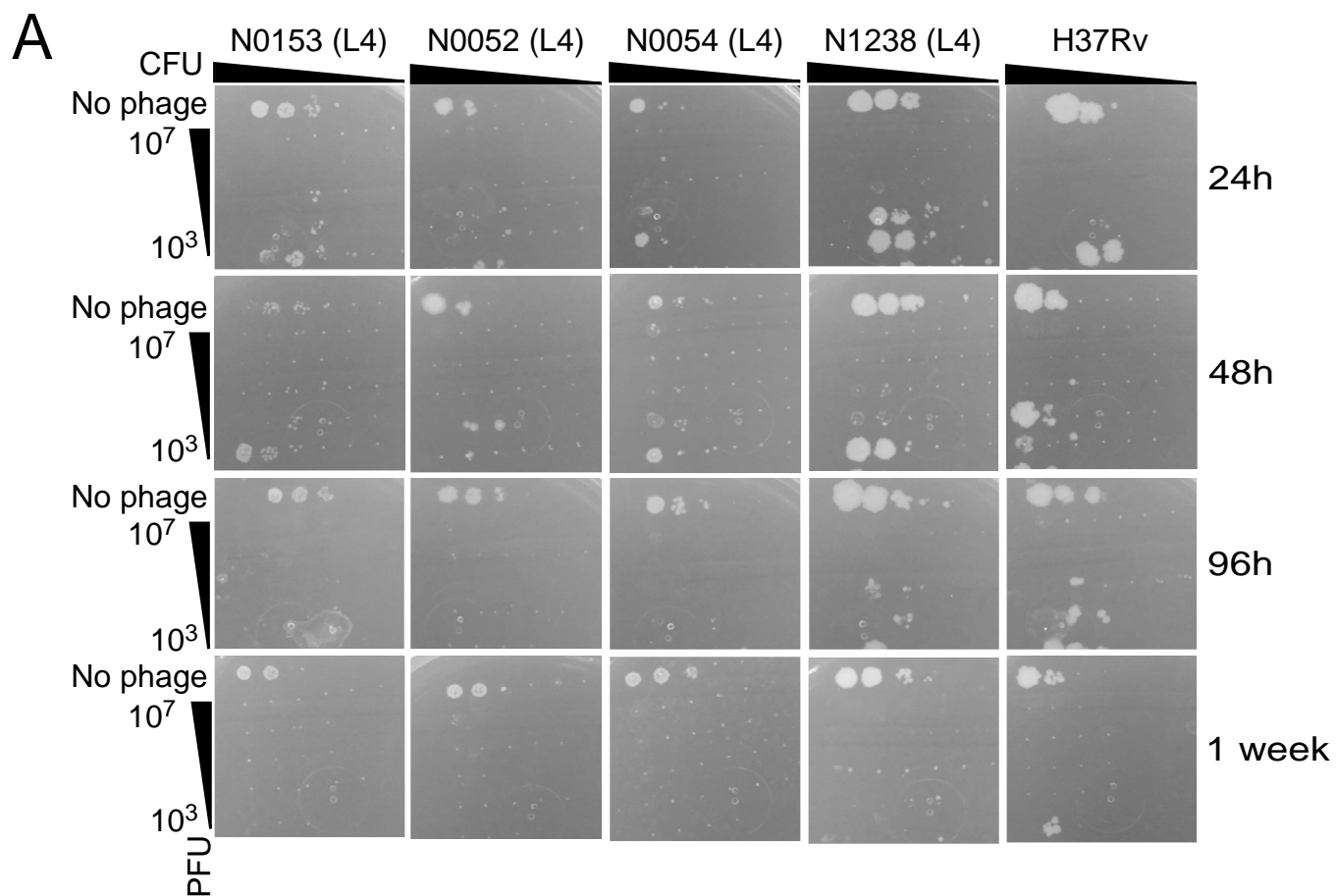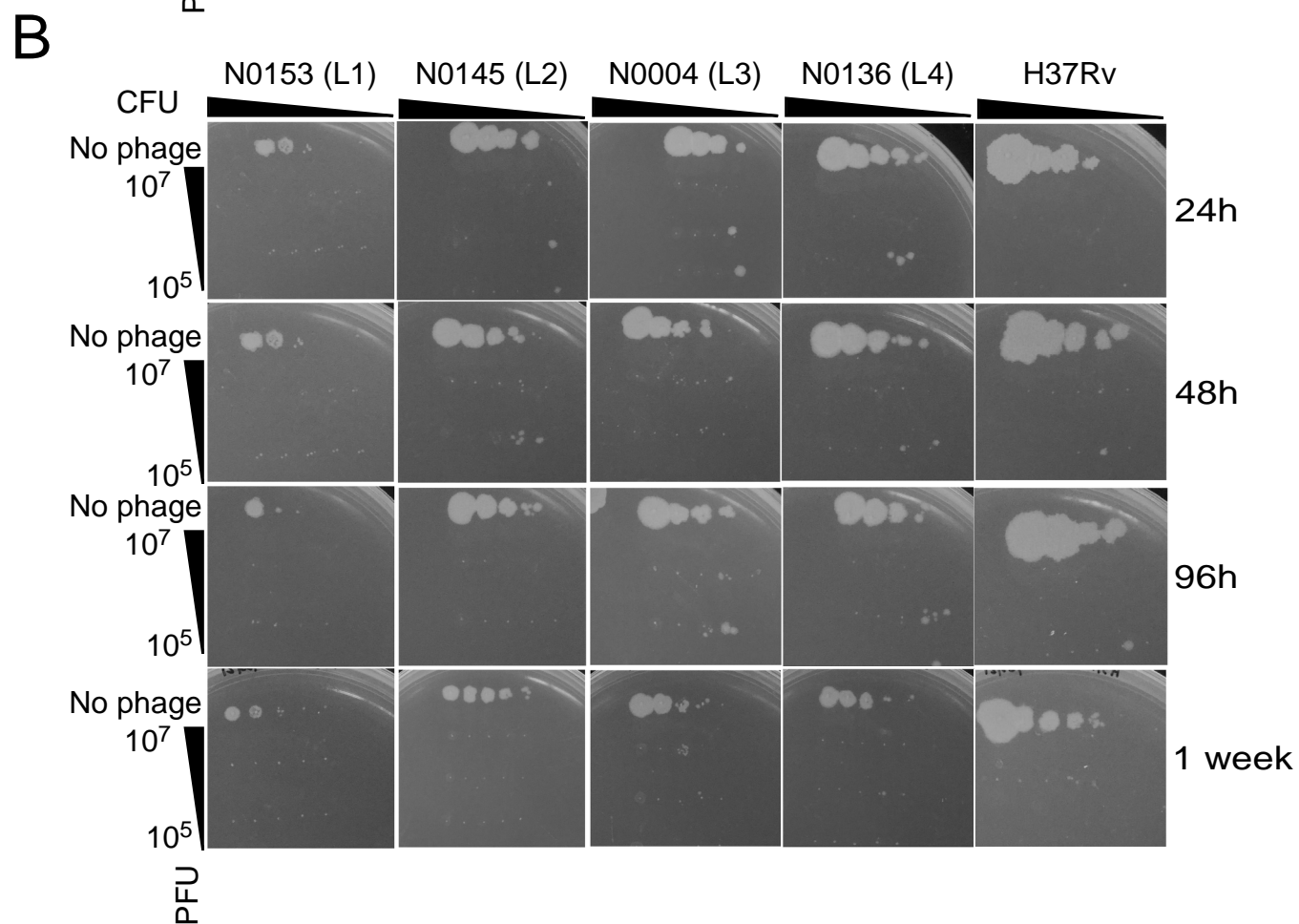

Figure S1

Supplement: FIG S1 [file mbio.00973-21-sf001.pdf]
